# Border Region Emergency Medical Services in Migrant Emergency Care

**DOI:** 10.1001/jamanetworkopen.2025.3111

**Published:** 2025-04-03

**Authors:** Christine Crudo Blackburn, Mayra Rico, Lauren Knight, Brandy Sebesta, Kirk Niekamp

**Affiliations:** 1 Department of Health Policy and Management, School of Public Health, Texas A&M University, College Station; 2 USA Center for Rural Public Health Preparedness, School of Public Health, Texas A&M University, College Station; 3 School of Public Health, Texas A&M University, College Station; 4 Department of Epidemiology, School of Public Health, Texas A&M University, College Station

## Abstract

**Importance:**

Focusing on the US southern land border only through a security lens minimizes the impact of security infrastructure and migrant health needs on local emergency medical services (EMS) clinicians.

**Objective:**

To explore the perceptions and experiences regarding the impact of migration on EMS clinicians in the communities of study.

**Design, Setting, and Participants:**

This qualitative study included in-depth interviews with fire department–based EMS clinicians in 3 Arizona communities on the Mexico border from June 23 to 27, 2024. Interviews were conducted 1-on-1 while clinicians were on shift. Participants were recruited with the help of fire department leadership in each fire department.

**Main Outcomes and Measures:**

This was an exploratory study designed to identify how EMS clinicians perceive the influence of migration in the study communities. Thematic analysis was conducted using inductive, latent coding.

**Results:**

The 67 participants were predominately male (62 [93%]), which is reflective of the EMS profession in the border region. Years of experience as EMS clinicians were fairly evenly distributed: 5 years or less (14 [21%]), 6 to 10 years (18 [27%]), 11 to 20 years (18 [27%]), and 21 or more years (17 [25%]). EMS clinicians in these communities reported serving multiple populations, often responding to complex calls, and experiencing limited downtime and mass casualty–like scenarios when there are high numbers of border crossings. Participants perceived that the local EMS system was strained, and occasionally overwhelmed, and that greater financial support from the federal government was necessary.

**Conclusions and Relevance:**

The findings of this qualitative study of EMS clinicians suggest that migration has a complex, multidimensional influence on EMS clinicians in the border region. Deterrence-focused actions have not decreased the number of crossings but rather pushed migrants to cross in more dangerous ways, leading to more injuries and deaths. Our findings suggest that the strain placed on local EMS clinicians is unsustainable and may be exacerbated by increased deterrence-based policies. Instead, border-region EMS clinicians need increased federal funding to support their work.

## Key Points

**Question:**

How do emergency medical services (EMS) clinicians along the US southern land border in Arizona characterize the impact of migration on their work?

**Findings:**

This qualitative study among 67 EMS professionals found that these clinicians serve multiple populations, often respond to complex calls, and experience limited downtime and mass casualty–like scenarios when there are high numbers of border crossings.

**Meaning:**

The findings of this study suggest that the strain placed on local border EMS clinicians is unsustainable and that further federal-level support is required.

## Teaser

This qualitative study among emergency medical services professionals in 3 Arizona communities on the border with Mexico examines how these clinicians perceive the impact of migration on their work.

## Introduction

Since 2021, encounters between migrants and US Customs and Border Protection (CBP) along the southern US land border have increased.^1^ This migration is often discussed within the security context, creating a singular focus on enforcement actions while overlooking the medical and emergency care requirements that accompany migration to the United States.^2,3^ These requirements, however, are substantial. For example, hospitals in San Diego experienced a 10-fold increase in border wall–related trauma between 2016 and 2021, and deaths among migrants entering the United States rose 162% from 2020 to 2022.^4^ While these numbers are significant on their own, they do not account for the numerous additional migrant-related calls attended to by local border region emergency medical service (EMS) clinicians. These calls range in severity and complexity from nonmedical or nonacute needs to childbirth and severe trauma as well as those who are dead on EMS arrival.

To date, there is limited examination of EMS provision along the US-Mexico border. Previous studies demonstrate that EMS clinicians in the border region experience higher call volumes, proportionately more trauma calls, and more obstetric emergencies and childbirths than their non–border region counterparts.^2,5,6^ Many of these differences are directly related to migration across the US-Mexico border, and many of the trauma calls that border-region EMS clinicians respond to are a direct result of the security infrastructure along the border.^7^

Focusing on the southern border through only a security lens creates a gap in understanding how migration affects the provision of EMS for both residents and migrants. A security focus also minimizes the influence of security infrastructure on medical professionals and first responders in communities along the US-Mexico border. To address this gap in understanding the humanitarian, public health, and medical needs related to migration, our study examined EMS clinician perceptions of and experience with the influence of migration on EMS provision in 3 Arizona border communities.

## Methods

To identify and characterize EMS clinician perceptions of the influence of migration on EMS provision, we conducted 67 in-person interviews with clinicians in 3 Arizona border communities. Interviews were conducted between June 23 and 27, 2024. All participants were employed at a local fire department.

### Ethics Statement

This study was approved as exempt by the Texas A&M University institutional review board. The research team received verbal consent from participants, which was captured on the audio recording. This study followed the Consolidated Criteria for Reporting Qualitative Research (COREQ) reporting guideline.

### Characteristics of the Study Team

There are 5 authors of the current study. Three authors identify as White females, 1 as a Hispanic female, and 1 as a White nonbinary person. No authors have certification or work experience in EMS, but 2 authors have extensive experience conducting qualitative research in the border region.

### Setting and Process

The study utilized naturalistic inquiry^8^ as the conceptual framework, and interviews were conducted in person in a 1-on-1 setting. In addition to interviews, the members of the research team conducted an ambulance ride along and traveled with fire department leadership to important points of interest for migrant-related emergency calls to conduct observations and gain contextual understanding. In community 1 and community 2, interviews were conducted at the fire station to which the participant was assigned. In community 3, interviews were conducted in the fire administration offices at city hall. The research team was provided with offices, conference rooms, and training rooms to ensure privacy during the interview process. Participants were interviewed only once, although 3 interviews had to be paused and restarted later because the participant had to respond to an emergency call. Interviews were audio recorded for accuracy, and handwritten notes were taken. The research team focused on achieving meaning saturation, which required a higher number of interviews.^9^

### Recruitment and Sampling

Participants for the study were recruited using purposive sampling. Using this sampling method, the research team intentionally selected participants who were currently working as emergency medical technicians, paramedics, or fire department leadership in the 3 study communities. During recruitment, only 2 individuals declined to participate.

### Data Analysis

The study was conceptualized and designed by C.C.B. Interviews were conducted by C.C.B., L.K., B.S., and K.N. Interviews were recorded and transcribed using NVivo transcription software version 14 (QSR International). Interview length averaged approximately 32 minutes. Once the audio was transcribed, the transcripts were quality checked by C.C.B., M.R., and L.K. Transcripts were not returned to participants for review.

Inductive coding of the data was conducted by C.C.B. During the first round of coding, latent codes were created.^10^ C.C.B. determined that meaning saturation was reached for all codes except one. The code that did not reach meaning saturation was “Border communities present unique challenges to EMS providers.” Once analysis was completed, the coding was reviewed by M.R. The final names of codes were adjusted to ensure they accurately reflected the data. Lastly, on completion of the analysis, fire department leadership in participating communities were contacted to review the findings of the study and offer feedback.

## Results

Participants were predominately male (62 [93%]), which is reflective of the EMS profession in the border region. Years of experience as EMS personnel were fairly evenly distributed: 5 years or less (14 [21%]), 6 to 10 years (18 [27%]), 11 to 20 years (18 [27%]), and 21 or more years (17 [25%]) (Table 1). We identified 3 primary themes, each of which contained several subthemes (Figure).

**Table 1.** Characteristics of Study Participants

Characteristic	Participants, No. (%) (N = 67)
Sex	
Male	62 (93)
Female	5 (7)
Years of experience	
≤5	14 (21)
6-10	18 (27)
11-20	18 (27)
≥21	17 (25)
Work location	
Community 1	19 (28)
Community 2	13 (20)
Community 3	35 (52)

**Figure.**
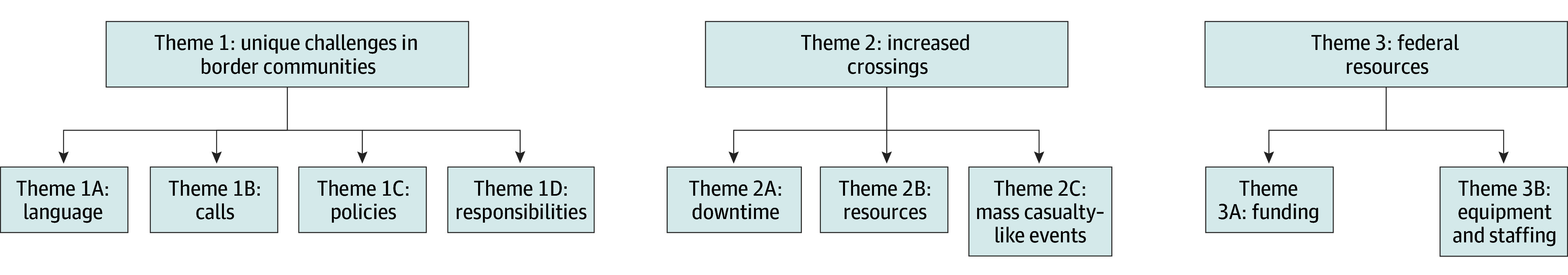
Coding Tree This provides a visual of the parent codes and child codes used as major themes.

### Theme 1: Unique Challenges for EMS

This primary theme spoke directly to the challenges that participants encountered that they believed were unique to serving a border community and were informed by 4 subthemes (Table 2). These subthemes included language barriers, call volume, policies, and responsibilities to residents as well as migrants.

**Table 2.** Challenges Reported By EMS Clinicians Working in a Border Community

Participant ID	Subtheme	Quotation
P3	Language barriers	“We had a one situation where some people from Senegal, I think it was Senegal, they speak French. They got shot, a group of them. So, when we got there, I asked them. I’m like, nobody knew who they were or anything. When I got one of them, [they] gave me a passport. It said Senegal. I Google. What language was Senegal? French. Google Translate it.”
P7	Language barriers	“Language barriers, because not all of them are Spanish speaking. I mean, we’ve seen, you name it: Brazilian, Russian, Arabic.”
P24	Language barriers	“Sometimes the language barrier is just unimaginable, I guess it would be the word. And even though we have the Google translator that can help us, as you see some of the areas where we’re at. There’s also there’s no cellphone coverage. So even if we wanted to use our Google translator, we wouldn’t be able to. So, it is challenging.”
P26	Language barriers	“I’ve been talking to Portuguese and Spanish. Just like a couple others somewhere like else so like Chinese. I think I got to pass out a couple of those. So, it’s a little bit of a challenge trying, dealing with these kind of... because you can’t even communicate. So, it’s mostly like signal hand signals and trying to figure everything out.”
P31	Language barriers	“The department is all about making it work like the fire service in general. You try and mime, you get, you know, okay, I’m sure that this word has got to be close enough in Spanish as it is to whatever your audience. Like, I’m hoping if I go Corazon and you go, thumbs up, we can start there.”
P34	Language barriers	“We don’t speak their language, it’s not even people from Mexico. You’re talking about South Americans, Brazilians, Haitian people, people from Haiti, from Russia, Asian people. We don’t understand the language. And it was stressful for us trying to assess them, making sure they were trying to correct their day, making their day better at the same time, which is that’s our job, to make their day better.”
P65	Language barriers	“Or they’re from a different parts of South America, you know, just to Spanish, different terminology, and especially with medical terminology. And uh, you know, it’s a really complicated talking to these patients, especially in a 911 setting where it’s a very serious call or it’s a respiratory call or chest pain call and you’re just thrown into a situation where you’re like, you have no communication with these people.”
P1	Higher call volume	“Definitely the call volume. It’s significantly higher in terms of EMS calls.”
P11	Higher call volume	“But our call volume has increased between 300 to 500 calls every year. So, we’re keep getting busier and busier because of the need from us...”
P38	Higher call volume	“I would say so, because over there you get a lot of calls because there’s a lot of people here there’s not that very many people but there’s a lot of calls.”
P57	Higher call volume	“The amount of calls to the border. It’s obviously crazy.”
P30	Calls from Mexico	“So, since they’re Americans, they call the Mexican Red Cross, which is their ambulance service. [The] ambulance brings them to the port of entry. We pick them up, they give us whatever pass down. And this poor gentleman was struck by a vehicle, both legs broken, not in casts. [It had] been like several days. [He] had internal injuries, had already lost the function of one kidney and was probably either already losing the second one or was close to it.”
P14	Complex calls	“There is a difference to the level of response. Just not the resources required. Because, you know, we always deal with multiple patients, just not with one or just multiple patients also.”
P48	Complex calls	“And so what you’re seeing is kind of this raw, unfiltered, you know, presentation of people who are coming from any variety of situations, whether that’s affluence or extreme poverty, you know, all the associated health outcomes that come with that, all the means that they might undertake in an effort to actually get here.”
P1	Infrastructure-related injuries	“But typically, if we’re going to the wall itself with border patrol, we’re more than likely going to be on a trauma. Usually it’s lower half of the body because that’s sometimes what hits first. But in calls something like that for traumas for the wall, where we’re in are like full spinal precaution mode, so back boards, c-collars, those are the type of calls that are—I’d probably say seven times out of ten—we’d run out of there from the wall.”
P2	Infrastructure-related injuries	“Well, for the border fence, like right now, it’s going to be a lot of, like, dehydration and a lot of falls; trauma because they fall from 40 feet, I think it’s a 40-foot wall... so there’s a lot of trauma.”
P18	Infrastructure-related injuries	“So, people, of course, they’re [the border fence] tall, like 30 to 50 feet those fences. And they’re trying to get up and down before the border patrol comes. And they use makeshift items. Sometimes they break, sometimes they don’t realize how high it is…. A lot of them fall. We went to one where one guy fell, broke his legs. The guy following him fell on his head and squished it. And the last guy is like, No, I’m not going down. So, we had to go take him down off the fence. He couldn’t get down.”
P30	Humanitarian needs	“And then we actually had coworkers that went and bough bread and stuff to make sandwiches and cases of water. And we took blankets and, you know, whatever we could really to kind of help out, especially in the beginning when there were so many people and they would be there for, like weeks, days with no food, no water. And it was hard too because a lot of times they weren’t even calling because they were sick. They were just hungry, you know.”
P65	Nonmedical or nonacute calls	“And the reason we knew this is because these people would tell us because they would call 911 because they don’t even know where to stay. And we’re like, hey, we don’t know where to go. It’s like, oh, all we can do is take you to the hospital. And then it’s hot. So they’re like, oh, we’re overheating. So we’ll take sometimes a group of 3 or 4 people to the hospital, an ambulance. And because they’re a family, you know.”
P47	Federal policies and politics	“And so I’ve lived up in a part of the country that it is, it’s out of sight, out of mind.”
P6	No transport capabilities in border patrol	“I would expect them to have like, um, like SWAT team level ambulances with like, ability to like, transport like, five people at the same time…. And like I, I would expect federal, like federal, I would expect them to have like a really secure vehicle but with like the ability to be doing like um medications and all that stuff. But no if it’s serious again they call us.”
P27	No transport capabilities in border patrol	“They’re certified. But they cannot transport via like ambulance, you know, so. They do just like a first aid first treatment and stabilize them…. They do respond to EMS call, but not as frequent as we do. And so and like I said, they’re only limited to, uh, to what they can do on scene, and then they transfer care to us.”
P30	No transport capabilities in border patrol	“Some of them were just border patrol agents, so they didn’t have any medical training and they had like an EMT or paramedic floating back and forth throughout the border handling medicals. But if it was something that was severe enough that needed to be transported, then they would obviously call us to step in.”
P36	No transport capabilities in border patrol	“It’s something beyond their capabilities, they’ll end up calling us.”
P47	No transport capabilities in border patrol	“Heat stroke, obviously, or [where] an immediate action is necessary. That’s where border patrol comes into play. However, the EMS services in that community are still responsible for receiving that patient from the border patrol medics and making and doing the transports, the ambulance transports and everything. So, it still falls on us, the EMS services in the community.”
P48	No transport capabilities in border patrol	“But it’s also an effort to, you know, they train many of them to EMT or and sometimes paramedic level. They’ve got their Borstar teams. They are acutely aware of the humanitarian dimension to this crisis. That said, there’s still ultimate reliance on the first responders inherent to the community. So, border patrol doesn’t transport patients. You know, they aren’t there, not necessarily, to my knowledge, able to affect a rescue off the border fence. There are circumstances that they are able to either render that initial care, but in most circumstances, it doesn’t absolve us of our responsibility to deliver and execute that EMS mission, regardless of citizenship status [or] anything else.”
P66	No transport capabilities in border patrol	“They [border patrol] have onsite [within the detention centers] nurses, onsite doctors, EMTs and paramedics that work there. And so, when a patient needs to be transported to a hospital, they deem the patient needs to be transported, we’re called. We are the transport agency in [community name].”

Abbreviations: EMS, emergency medical services; EMT, emergency medical technician; ID, identification; SWAT, special weapons and tactics.

#### Subtheme 1A: Language Barriers

Many of the EMS clinicians interviewed for this study are fluent Spanish speakers, but the sheer variety of languages that they encounter meant they often treated a patient that was speaking a language they did not understand. The primary way in which participants overcame this barrier was by using a web-based real-time translator. In some cases, they did not have cellphone service to use the app or could not determine what language the patient was speaking, and even in the best-case scenario, using the app slowed care provision and limited the amount of information they could obtain from the patient.

#### Subtheme 1B: Call Volumes and Types

Most participants discussed both the number of calls that they received given the size of the community and the uniqueness or complexity of those calls. Participants from community 1 in particular noted that many of the calls came from US citizens living in Mexico and these patients often had severe trauma or advanced illness. Additionally, many participants described how border security infrastructure affected their call volume and the types of injuries that they respond to. The trauma caused by falls from the top of the border wall was one of the most common migrant-related calls they responded to, but many participants also spoke of the uniqueness of addressing the humanitarian needs of their patients, such as providing food, water, and blankets. The complexity of calls and unique challenges for EMS clinicians in these border communities was summed up by participant 6, who said, “There’s no playbook for how to handle going in there. You have to use your common sense.”

#### Subtheme 1C: Federal Policies and Politics

Many participants discussed the ways that they are affected by federal policies. Participant 48 described their frustration with the federal policies and politics saying,

The challenge is [that] this issue is inextricable from the political dimension that’s attached to it.... So, we’re caught in this middle ground of: this is too convenient of a political problem to go to waste or we don’t want to acknowledge the gravity of this problem because then it forces us to have to solve it or deal with it.... And I think that to them [politicians on both sides of the aisle], this is just a political football. To us this is something where it has profound effects...

The feeling that federal policies and federal decision-makers on both sides of the aisle ignored the influences of immigration policy on the local community was consistent across participants.

#### Subtheme 1D: Responsibilities to Migrants and Residents

Participants were mostly in agreement that they had a positive relationship with border patrol agents and that agents often helped EMS clinicians by providing basic care, controlling the scene, or providing access to the patient. Importantly, however, local EMS was responsible for all patient transports. Therefore, while border patrol has some medical capabilities both in the field and within the detention centers, local EMS is the only certified transport agency (Table 2).

### Theme 2: Experiences of Increased Number of Border Crossings

Times of increased border crossings created different changes. The subthemes included impact on downtime and resources (Table 3).

**Table 3.** EMS Clinicians’ Experience of Increased Migration

Participant ID	Subtheme	Quote
P8	Reduces or eliminates downtime	“Because we were going back and forth one call after another after another after another after another. No downtime. No, we get water, but sometimes we had our breakfast at 8 pm because it got that busy.”
P32	Reduces or eliminates downtime	“You know, almost as if, you know, one ambulance was passing the other, you know?”
P52	Reduces or eliminates downtime	“They run constantly anywhere from 16 to 20 calls a day. Like constantly. There’s only 24 hours in a day, so put it together.”
P55	Reduces or eliminates downtime	“So your downtime becomes a little more infrequent during the day, which also affects your opportunities to write reports for the calls you’ve done. And keep the calls separated in your mind. Because your calls can stack. Did I start an IV in his left arm? No that was hers. I gave her an IV and she had shortness of breath. He had chest pain. And you start to crisscross your calls at times, of course. Now, let’s get into the personal side of it. I’m thirsty. I’m hot and sweaty. I’m hungry.”
P62	Reduces or eliminates downtime	“It’s just if you get a call, you’re going. You know, so a lot of times you’re doing standup 24 hours and grabbing lunch from the hospital or grabbing a sandwich or something using the bathroom there and then getting back out on a call.”
P4	Depletes available resources	“Now, you do have ambulances. In, that first we were getting depleted or whatever. So now someone has a heart attack here in the city. So now we’ve got to wait 25 minutes to get an ambulance because those resources got depleted…. It’s tough.”
P14	Depletes available resources	“And it gets overwhelming, you know, because we deplete all our ambulances, all our resources, and we kind of tend to the people that we’re supposed to be protecting. Yeah, it does overwhelm us.”
P47	Depletes available resources	“We run on limited budgets for our own communities. You know, we run about as thin as possible to take care of the needs of our communities and this, you know, and I hate using the word burden because these are human lives, but it does add an extra burden to what we’re already doing.”
P14	Creates mass casualty–like scenario	“And that’s the thing when you have 30 or 40 migrants, I mean, I don’t have time to deal with one. It’s either, ‘Okay, who needs help?’ It’s a triage situation. We’re already overwhelmed. I mean, I’m going to take whoever needs it the most.”
P22	Creates mass casualty–like scenario	“Yeah, cause everybody was getting overwhelmed. Us, the surrounding agencies, border patrol is getting overwhelmed. We responded to the levee, I think it was from 2 to 3 in the morning. We didn’t see border patrol. They were saying that they were sending someone over, they were going to send a bus or whatever. And we had a group of 150, 200 people and they just see us pulling up and they just kind of swarmed us and we just, we never know what’s going to happen in those cases because there’s so many of them and there’s only two, three, four of us on scene.”

Abbreviations: EMS, emergency medical services; ID, identification; IV, intravenous.

#### Subtheme 2A: Clinician Downtime

Most participants discussed how the call volume made it impossible for them to eat, sleep, or even find time to use the restroom. The feeling of running back-to-back calls for an entire shift—something that participants referred to as a standing 24—became normal when crossing levels increased. Participants stressed that this did not happen on just one shift, but that it occurred on every single shift for the duration of the increase. Thinking back to the most recent increase in crossings that had occurred in the community, participant 19 said, “We probably had about 20 to 25 calls a day. And it was continuous.”

#### Subtheme 2B: Resources

Many participants expressed concern that the diversion of resources to the border left community residents without timely emergency medical access. Participants recognized that providing care for large numbers of individuals above and beyond the population that determines their funding and resources presented challenges and often left them at risk of not having resources available for the local community.

#### Subtheme 2C: Mass Casualty–Like Scenarios

One of the most interesting dimensions of the influence of increased crossing was the way in which it created mass casualty–like scenarios for the participants in our study. Mass casualty events are those that have so many people needing care that it overwhelms EMS and the local health care system. Within disaster and emergency planning, EMS clinicians are given specialized training to deal with the unique and high-stress environment of a mass casualty event. For participants in our study, increased numbers of crossings created mass casualty events that occurred day after day for months. Participant 11 described arriving to a call during this time,

Right now this person’s having difficulty breathing. This one was [*sic*] having a baby any minute now. This difficulty breathing one, which is they have asthma. They have an inhaler. Their problem’s worse than this one. And the baby’s coming.

Participant 30 reported a similar experience:

So, when we would get there, and you would see hundreds [of people].... And it was kind of like a little revolving door for a while, you know, just multiple people. And you would just have to figure out who’s the worst, who needs to go now first, you know.

Mass casualty events are widely recognized as unique and challenging scenarios that require specialized training and skills.^11^ These events are rare, and many EMS professionals never encounter one. For the border-region EMS clinicians in our study, however, times of increased crossings created numerous, continuing, mass casualty-like events (Table 3).

### Theme 3: Needed Support

The final primary theme was that the EMS clinicians and, by association, the fire departments they are nested in need more resources. Representative quotations appear in Table 4.

**Table 4.** Resources Needed by EMS Clinicians to Care for Migrants

Participant ID	Subtheme	Quote
P3	Need more federal support	“So, they’re getting, you know, the federal government understands that they need that help and they’re getting that funding. But local communities are being left aside. Not getting any of that funding. Not getting any of the help. My biggest thing is that on the law enforcement side, you do have help…. But there is nothing on the EMS fire side of that where the federal government is understanding that, hey, the resource, we are eating up these resources from local communities.”
P6	Need more federal support	“Like that’s federal like they can, you know, they spend like millions and millions I want to say billions. Billions on the construction of the border [infrastructure]... it takes a little bit of money and like, you can get, like, four more ambulances.”
P7	Need more federal support	“I believe the federal government, you know, they should help us out. Our police department, they have this, it’s called Stonegarden. [It’s] Federal funding. We should have something like that here.”
P48	Need more federal support	“But at the end of the day, it was kind of a shrug of the shoulders of what we’re experiencing and what our needs were. And so that’s kind of that lends itself to more or less the general perception that I think exists, which is kind of this abandonment that border communities have by the federal government…. But you [would] think I was asking for the first born child, it’s like I’m just asking you for the resources to do our job effectively.”
P53	Need more federal support	“I think that the federal government thinks that this is, it’s a state and local problem. And that we’re going to go back to money here. You know, it’s just [that] the state and local entities cannot support day after day after day after day [of nonresident care]. At some point in time, this is a federal problem.”
P8	More ambulances and clinicians	“We need more for cases like that when it gets... there’s lot we need. Either overtime because we need manpower. It’s not just the ambulances. We need to man them up and we need more because it’s overwhelming. It’s a lot.”
P53	More ambulances and clinicians	“We went from a department that was generally seeing, you know, 14, 15 000 calls. And it’s just it’s just increasing year after year. But personnel is not increasing. Equipment is not increasing.... And so the system at some point in time breaks down.”
P4	Need better access	“Yes but sometimes there wasn’t access. It got to the point where I talked to the public work guys, and they have like a basket, like a little smaller truck with a basket once a use for APS. So I think we started considering using that one to just to help us get people that were I don’t know if they were getting stuck if they were being afraid, but we do.”
P60	Need better access	“A challenge, and getting from almost, like, where a staging area is, I guess, we had to get in the border patrol agent’s truck. He’s like, well, I only got two seatbelts. I can only take two.”

Abbreviations: APS, Arizona Public Service; EMS, emergency medical services; ID, identification.

#### Subtheme 3A: Federal Funding

All participants in the study believed that the federal government should be the one to provide the additional resources needed. They noted that the local law enforcement agencies were supported through a federal program, which allowed them to purchase more equipment and resources as well as pay overtime for clinicians, and participants believed that the federal government should provide something similar for fire departments and EMS. Participants who served in administrative roles said that there was some reimbursement if the individual was in border patrol custody, but this reimbursement was only at the Medicaid rate and was often slow to receive, leaving the local departments to cover large uncompensated costs. Participant 59 expressed this frustration, stating, “I feel [like] we’re serving a federal issue at a municipal level and without the support or without the finances or without the resources.”

#### Subtheme 3B: Equipment and Staffing

Participants believed that their department needed more ambulances and funding to support additional clinicians to meet the needs of the local population, US citizens coming to the port of entry, seasonal tourists, and migrants crossing the border. Participants reported that they often had to rely on border patrol agents to help them reach patients in the remote desert because they lack off-road vehicles and that this reliance on border patrol often meant that they could not take all the necessary medical supplies with them (Table 4).

## Discussion

Our findings show that fire department–based EMS clinicians in the 3 study communities face unique challenges, experience impacts on themselves and resources due to border crossings, and need more resources to meet the demands creating by serving community residents, migrants, and US citizens residing in Mexico. Participants in the study emphasized that they experience higher call volumes than similar communities their size because they serve multiple populations (ie, residents, the migrant community, and US citizens residing in Mexico). Additionally, our findings confirm and deepen understanding of previous research related to the effects of border security infrastructure on local EMS professionals.^2,7^ Our findings also highlight differences between EMS provision in the border region and EMS provision at large. Most notably, the consistent need to overcome language barriers, even for EMS clinicians who speak Spanish, and regularly being faced with mass casualty–like scenarios—sometimes for multiple shifts in a row and for numerous months without a break—further compounds industry-wide challenges for EMS in the border region compared with their non–border region counterparts.

In addition to deepening the understanding of border infrastructure influences, our study highlights the reliance on local EMS clinicians for transport of migrants both at the border and within detention centers. To date, there is no literature of which we are aware that examines US CBP’s reliance on local EMS to transport migrants, and our study finds that even in instances where border patrol agents can offer initial care, local EMS agencies remain the only option for patient transport. This reliance on services without adequate resource support creates a strain on the local EMS system. While there is no previous literature that speaks to these findings, the US CBP Implementation Plan for Enhanced Medical Support Efforts published in 2020 states, “USBP shall rely heavily on referral to local health systems.”^12^ Thus, the reliance on local EMS services that was observed in this study is likely the result of CBP medical plan implementation.

While EMS clinicians are the first to encounter sick and injured migrants, particularly those with severe trauma must be transported to local hospitals, placing strain on local physicians and hospital systems as well. Additionally, the US focus on deterrence-based policies, such as adding more border fence or reducing the ability of people to apply for asylum, will not address the challenges faced by the participants in the study or other border-region EMS clinicians facing similar issues. Such deterrence-focused actions have not decreased the number of crossings^15^ and have, instead, pushed migrants to cross in more hazardous places and in more dangerous ways leading to more injuries and deaths.^4,16^

We found that the participants in this study generally did not take issue with providing care to migrants. They viewed the provision of this care as an important part of their job duties. They did, however, believe it was unrealistic to ask them to provide care to both residents and migrants with the resources estimated to serve only the local community. Previous research has shown that EMS clinicians across the country are facing higher demands for their services^13^ and these demands can lead to higher rates of burnout.^14^ These well-established findings suggest that the higher call volumes among border EMS clinicians combined with insufficient financial, equipment, and clinician support for the demands of the border region could lead to higher stress and burnout levels. Therefore, understanding the needs of these EMS professionals is vital for protecting emergency access to care for residents and migrants alike.

### Limitations

Our study has several limitations. First, the findings are not generalizable to other EMS professionals in other border communities. The challenges facing border communities in Texas and California may differ due to factors such as crossing volumes and local and state resources. Additionally, due to the small, rural nature of 2 of the study communities, it is possible that the influences of migration were felt more acutely. Further research is needed to examine the similarities and differences between border communities by conducting a multistate, mixed-methods study. This will be necessary to develop a more thorough understanding of the influence of migration on EMS clinicians and border communities. Despite these limitations, to our knowledge, this study provides the first examination of the influence of migration on rural and small city border communities and provides foundation knowledge about how migration affects border-region EMS clinicians.

## Conclusions

In this qualitative study of EMS professionals, our findings demonstrated that migration has a complex, multidimensional influence on EMS clinicians in the border region. Challenges reported by clinicians included language barriers that complicated fast and efficient care provision, infrastructure-related injuries that resulted in severe trauma, and reliance on local EMS clinicians for migrant care without adequate additional resources. Based on existing data, it can be hypothesized that moving to strengthen border infrastructure and security is likely to increase the current burden on border-region EMS clinicians and professionals. Therefore, our findings suggest that the strain placed on local EMS clinicians is unsustainable and may be exacerbated by increased deterrence-based policies. Instead, border-region EMS professionals need increased federal funding to support overtime pay, additional physical resources, additional staffing, and the ability to meet the humanitarian needs they are frequently required to address.

## Notes

Supplement.
Data Sharing Statement
