# Supplementary material for: Border Region Emergency Medical Services in Migrant Emergency Care
Source: JAMA Netw Open. 2025 Apr 3;8(4):e253111. doi: 10.1001/jamanetworkopen.2025.3111 (PMC11969281; doi:10.1001/jamanetworkopen.2025.3111)
Supplement: Supplement. — Data Sharing Statement [file jamanetwopen-e253111-s001.pdf]

Data Sharing Statement

Blackburn. Border Region Emergency Medical Services in Migrant Emergency Care. *JAMA Netw Open*. Published April 03, 2025. doi:10.1001/jamanetworkopen.2025.3111

Data
Data available: No

Additional Information
Explanation for why data not available: Data will be available upon reasonable request, but some redaction will be needed to protect participant confidentiality.
